# Prescribed time backstepping sliding mode control for attitude stabilization of plant-protection UAVs under wind and motor disturbances

**DOI:** 10.3389/fpls.2025.1689665

**Published:** 2025-12-03

**Authors:** Chenqi Zhu, Zhikai Wang, Dongkai Zhang, Pengju Si

**Affiliations:** School of Information Engineering, Henan University of Science and Technology, Luoyang, China

**Keywords:** prescribed time convergence, extended-state observer, backstepping sliding mode, numerical simulation, uniform spraying

## Abstract

In this paper, a novel prescribed time convergent backstepping sliding mode control method for robust attitude stabilization of plant-protection UAV is proposed to solve the uneven pesticide spraying issue caused by strong gusts and motor dynamics. Firstly, a mathematical model of the plant-protection UAV with disturbance is established, which regards the gust and the dynamic characteristics of the motor as the total disturbances of the system. Secondly, a prescribed time extended state observer is proposed to accurately estimate the error. Subsequently, an integral sliding surface is proposed on which the states converge to the origin in prescribed time. Moreover, a prescribed time control method is proposed by combining the variable coefficient exponential approach law and the observer. Finally, the stability of the algorithm is verified by the Lyapunov functions. The numerical simulation results show that under different initial states, this method can achieve attitude stability in preset time, which provides a guarantee for the uniform spraying of the plant-protection UAVs.

## 1 Introduction

With the opening of low-altitude airspace and the rapid development of modern agricultural technologies, plant-protection UAVs have become vital tools for crop pest control due to their high efficiency, precision, and operational flexibility. Compared with traditional manual spraying or ground-based machinery, these UAVs can adapt to complex terrains, reduce pesticide waste, improve operational efficiency, and minimize health risks to the operators (Xie et al., 2022 and Gunasekaran et al., 2023). As one of the core technologies of plant-protection UAVs, attitude control stability directly affects spraying uniformity, coverage quality, and operational safety—particularly in terms of disturbance rejection and dynamic response performance in complex farmland environments. However, during actual spraying operations, especially in dynamic flight under complex conditions, factors such as strong wind disturbances, motor dynamics, payload variations, and air frame vibrations can easily cause attitude deviations. These deviations may lead to spraying inaccuracies, resulting in missed or overlapping spray areas, and, in severe cases, may even cause crashes, leading to significant losses (Hu et al., 2022; Li and Gong, 2023).

In recent years, internal and external scholars have conducted extensive research on attitude control for plant-protection UAVs. Chu (2024) achieved stable flight through a dual-loop structure combining outer-loop position control and inner-loop attitude control. Song et al. (2025) proposed an incremental attitude command generation method not limited to equilibrium flight modes, which significantly improved the UAV’s response capability to acceleration commands. Although traditional control methods are mature in plant-protection UAV applications, they still exhibit limitations in handling strong non-linearity, high coupling, and complex disturbances. The active disturbance rejection attitude control method proposed significantly enhanced control performance by improving the traditional ADRC architecture and incorporating a multi-stage feed-forward mechanism, reducing the attitude recovery time under gust disturbances to less than 0.2 s (Chen et al., 2025). Han et al. (2025) combined terminal sliding mode control with super-twisting sliding mode control to propose a dual-loop position-attitude control architecture for hexacopter UAVS that reduced machine vibrations. Yu and Xing (2023) considered trajectory tracking during rotor speed variations and introduced an extended state observer to design a composite sliding mode control law, enhancing the stability of the UAV system. To address the challenge of maintaining accurate altitude and attitude control under multiple actuator failures, Li et al. (2024) developed an adaptive robust fault-tolerant control algorithm for plant-protection UAVs, significantly improving the precision of the attitude tracking system.

Jin et al. (2024) addressed the issues of poor adaptability and weak disturbance rejection in the position-attitude control system of heavy-load quadrotor plant-protection UAVs by designing a fuzzy PID controller based on an improved genetic algorithm, thereby enhancing the attitude stability of spraying UAVs. Zhao et al. (2020) and Lian et al. (2015) introduced a non-singular terminal sliding mode attitude tracking control scheme, eliminating the algorithm’s singularity. Furthermore, Zhao et al. (2023) developed composite fast non-singular terminal sliding mode control schemes utilizing disturbance observer (DOB) technology. Nevertheless, the convergence time of these control methods still depended on the system’s initial state. To address this limitation, Jiang et al. (2023) proposed a fixed-time convergent attitude control method. Wang et al. (2025) presented a predefined-time convergent attitude control algorithm and ensured that the stabilization time is independent of both the system’s initial conditions and controller parameters.

To prevent the attitude stabilization of plant-protection UAV from being affected by the system’s initial conditions and controller parameters, addressing the uneven pesticide spraying issue caused by strong gusts and motor dynamics, this article aims to conduct research in the following aspects: (i) establish a mathematical model which considers wind gusts and motor dynamics as the total disturbances, (ii) integrate a prescribed time extended state observer (PTESO), (iii) design a predefined-time backstepping sliding mode control (PBSMC) for UAV attitude stabilization, and (IV) validate the scheme through numerical simulations.

## 2 Problem formulation

### 2.1 Attitude model of plant-protection UAV

**Figure 1:**
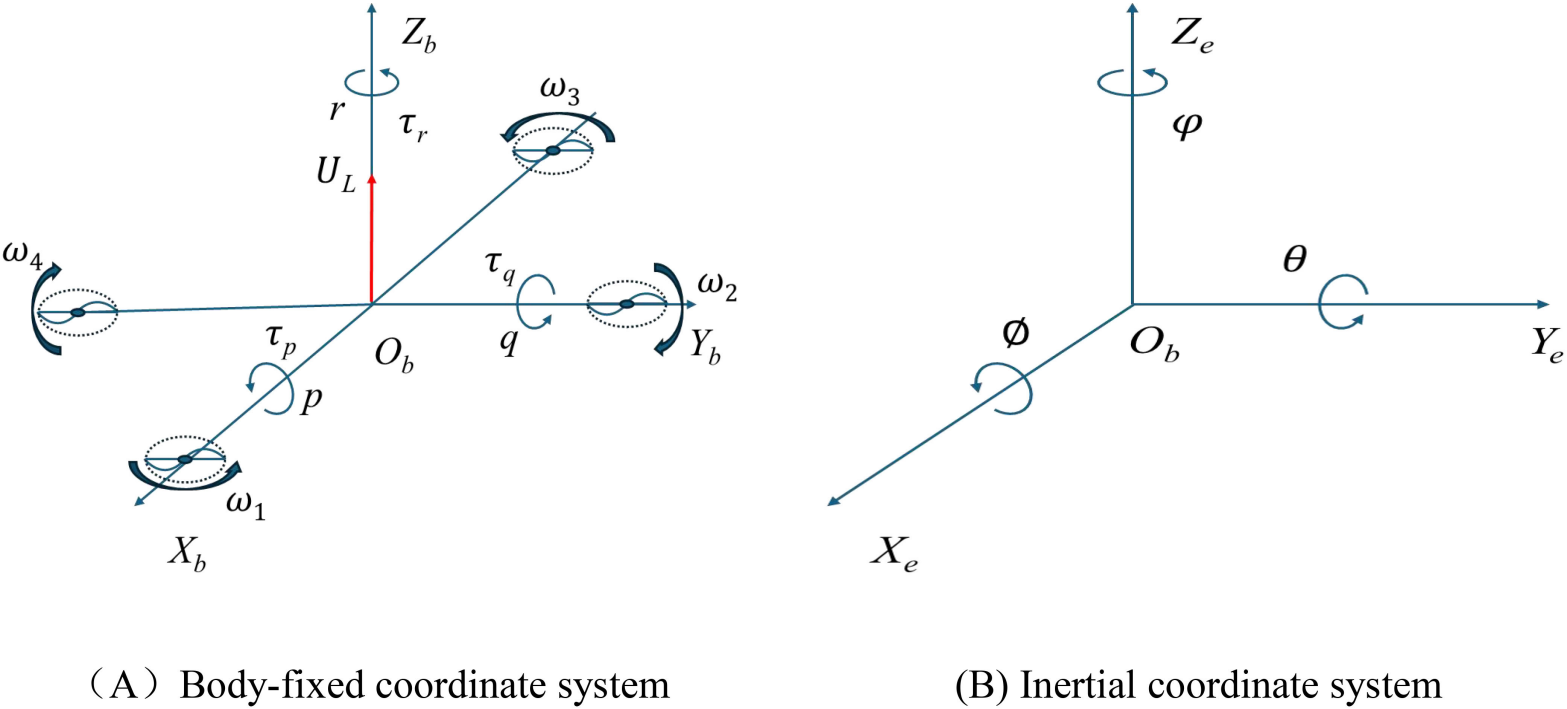
Schematic of the body-fixed **(A)** and inertial **(B)** coordinate systems.

The rotors of a quadcopter UAV are typically arranged in either a cross configuration or an X configuration. This paper focuses on a quadcopter plant-protection UAV with a cross rotor layout, whose rotor structure is illustrated in **Figure 1**.

In this figure, $O_{b}-X_{b}Y_{b}Z_{b}$ is the body-fixed coordinate system and the $O_{e}-X_{e}Y_{e}Z_{e}$ is inertial coordinate system. ϕ, Θand φ are the roll, pitch, and yaw of the UAV. p, q, and r represent the components of the UAV angular velocity vector projected onto the body-fixed coordinate system. The magnitudes of the rotational speeds for the four rotors are denoted as $\omega_{1}$, $\omega_{2}$, $\omega_{3}$, and $\omega_{4}$, respectively. The variable $U_{L}$ denotes the total lift force acting along direction $O_{b}Z_{b}$, while $\tau_{p}$, $\tau_{q}$, and $\tau_{r}$ represent the rotational moments (or torques) about the three axes of the body-frame system. Their mathematical expressions are given by Equation 1.

(1) $$\left\{ \begin{array}{l} U_{L}=k_{L}(\omega_{1}^{2}+\omega_{2}^{2}+\omega_{3}^{2}+\omega_{4}^{2})\\ \tau_{p}=lk_{L}(\omega_{2}^{2}-\omega_{4}^{2})\\ \tau_{r}=lk_{L}(-\omega_{1}^{2}+\omega_{3}^{2})\\ \tau_{r}=b(-\omega_{1}^{2}+\omega_{2}^{2}-\omega_{3}^{2}+\omega_{4}^{2}) \end{array}\right.$$

where $K_{L}$ is the lift coefficient, b is the anti-torque coefficient, and l is the distance from the rotor center to the UAV’s center of mass. The attitude dynamics model of the UAV is then given by Equation 2.

(2) $$\left\{ \begin{array}{l} \dot{\Theta }=W\Omega \\ \dot{\Omega }=-J^{-1}(\Omega \times (J\Omega ))+J^{-1}\tau +\tau_{d} \end{array}\right.$$

The mathematical expressions of the respective parameters are given below


$$\Theta =\left[\begin{array}{c} \phi \\ \theta \\ \varphi \end{array}\right],\Omega =\left[\begin{array}{c} p\\ q\\ r \end{array}\right],\tau =\left[\begin{array}{c} \tau_{p}\\ \tau_{q}\\ \tau_{r} \end{array}\right],J=\left[\begin{array}{ccc} J_{x} & 0 & 0\\ 0 & J_{y} & 0\\ 0 & 0 & J_{z} \end{array}\right],W=\left[\begin{array}{ccc} 1 & \sin\phi \tan\theta & \cos\phi \tan\theta \\ 0 & \cos\phi & -\sin\phi \\ 0 & \sin\phi /\cos\theta & \cos\phi /\cos\theta \end{array}\right],\tau_{d}=\left[\begin{array}{c} \tau_{d}^{p}\\ \tau_{d}^{q}\\ \tau_{d}^{r} \end{array}\right]$$

where $\dot{\Theta }$ is the derivative of Θ, and $J_{x}$, $J_{y}$, $J_{z}$, $\tau_{d}^{p}$, $\tau_{d}^{q}$, $\tau_{d}^{r}$ represent the moments of inertia about the body-fixed axes and the external disturbance torques, respectively. During UAV pesticide spraying operations, the condition of force equilibrium in the vertical direction is satisfied, thus yielding Equation 3.

(3) $$U_{L}\cos\phi \cos\theta -mg=0$$

where m represents the mass of the UAV, and g denotes the gravitational acceleration. The attitude angle tracking error variables can be obtained by Equation 4.

(4) $$E_{\Theta }=\Theta -\Theta^{d}=\left[\begin{array}{c} \phi -\phi^{d}\\ \theta -\theta^{d}\\ \varphi -\varphi^{d} \end{array}\right]=\left[\begin{array}{c} e_{\phi }\\ e_{\theta }\\ e_{\varphi } \end{array}\right]$$

The UAV dynamics model with attitude angle errors as state variables can be obtained by Equation 5.

(5) $$\{\begin{array}{l} {\dot{E}}_{\Theta }=W\Omega -{\dot{\Theta }}^{d}\\ \dot{\Omega }=-J^{-1}(\Omega \times (J\Omega ))+J^{-1}\tau +\tau_{d} \end{array}$$

By setting $X_{1}=E_{\Theta },X_{2}=\Omega ,D_{1}=-{\dot{\Theta }}^{d}={\left[\begin{array}{ccc} d_{11} & d_{12} & d_{13} \end{array}\right]}^{T},D_{2}=\tau_{d}={\left[\begin{array}{ccc} d_{21} & d_{22} & d_{23} \end{array}\right]}^{T},F=-J^{-1}(\Omega \times (J\Omega ))$, the simplified attitude error dynamics of the agricultural UAV are derived as follows

(6) $$\{\begin{array}{l} {\dot{X}}_{1}=WX_{2}+D_{1}\\ {\dot{X}}_{2}=F+J^{-1}\tau +D_{2} \end{array}$$

### 2.2 Preliminary definitions and lemmas

Definition 1. Consider the continuous nonlinear system described by Equation 7

(7) $$\dot{x}=f(t,x(t))+u$$

where $x(t)\in R^{m}$ x(t) is the system state vector, $u\in R^{m}$ denotes the control input, and f(•) is a nonlinear function characterizing the system dynamics with the constraint f(t,0)=0. The system is said to achieve prescribed-time convergence if, by designing an appropriate control input u, the system state x(t) converges to zero within any prescribed settling time T.

Lemma 1 (Ren et al., 2021)^17^. Consider the nonlinear system described by Equation 7. Let V(x(t),t)be a positive definite and continuously differentiable Lyapunov function satisfying V(0,t)=0. For all t=[0,∞), if there exist constants b≥0and k>0,which can make the time derivative of V(x(t),t)along the system trajectories satisfies $\dot{V}(x(t),t)\leq -bV-k\varphi (t_{0},T)V$, then Equation 8 holds.

(8) $$\{\begin{array}{ll} V(x(t),t)\leq -\mu^{-k}(t_{0},T)\exp^{-b(t-t_{0})}V(t_{0}), & t\in \left[t_{0},T\right)\\ V(x(t),t)\equiv 0, & t\in \left[T,\infty \right) \end{array}$$

where $\mu (t_{0},T)$ denotes a time-varying scaling function which is defined by Equation 9.

(9) $$\mu (t_{0},T)\{\begin{array}{ll} 1 & t\in \left[0,t_{0}\right)\\ {\left(\frac{T}{T+t_{0}-t}\right)}^{p} & t\in \left[t_{0},T\right)\\ 1 & t\in \left[T,\infty \right) \end{array}$$

where p, $t_{0}$and T are positive parameters constrained by p>1, $t_{0}\geq 0$and T>0. The first-order time derivative of $\mu (t_{0},T)$ is given by Equation 10.

(10) $$\dot{\mu }(t_{0},T)\{\begin{array}{ll} 0 & t\in \left[0,t_{0}\right)\\ \frac{p}{T-t_{0}}\mu {\left(t_{0},T\right)}^{1+\frac{1}{p}} & t\in \left[t_{0},T\right)\\ 0 & t\in \left[T,\infty \right) \end{array}$$

Furthermore, we define a function $\varphi (t_{0},T)$ associated with the scaling function $\mu (t_{0},T)$and its time derivative $\dot{\mu }(t_{0},T)$ as Equation 11.

(11) $$\varphi (t_{0},T)\{\begin{array}{ll} \frac{p}{T-t_{0}} & t\in \left[0,t_{0}\right)\\ \frac{\dot{\mu }(t_{0},T)}{\mu (t_{0},T)} & t\in \left[t_{0},T\right)\\ \frac{p}{T-t_{0}} & t\in \left[T,\infty \right) \end{array}$$

## 3 Prescribed time attitude control for plant-protection UAVs

Based on the attitude error dynamics given in Equation 6, this section proceeds as follows: First, two prescribed time convergent extended state observers (ESOs) are designed to accurately estimate the lumped disturbances *D_1_* and *D_2_* accurately. Then, a prescribed time convergent integral sliding surface is formulate and integrated with a variable-coefficient exponential reaching law to synthesize a prescribed time backstepping sliding mode control (PTSMC) law. Finally, the stability of the proposed control scheme is rigorously analyzed via Lyapunov theory.

### 3.1 Prescribed time extended state observer

Traditional sliding mode control (SMC) relies on fixed gain terms to compensate for system disturbances typically. While large disturbances necessitate correspondingly large fixed gains, this approach inherently induces severe chattering phenomena. To address this issue, this section integrates the state estimation error with the residual term encompassing the lumped system disturbance into a novel extended state, thereby constructing a prescribed time extended state observer (PTESO). Within this new framework, the fixed gain is only required to compensate for the observation error. By developing a well-designed observer that guarantees sufficiently small estimation errors, a reduced fixed gain can be employed, ultimately mitigating system chattering.

Regarding the attitude error dynamics of the agricultural UAV described by Equation 6, we introduce the following assumptions:

Assumption 1. The external disturbance *d(t)* is continuously differentiable, and its time derivative is bounded by a known constant $k_{dp}>0,k_{dq}>0,k_{dr}>0$which is shown in the Equation 12.

(12) $$\left|{\dot{\tau }}_{d}^{p}\right|\leq k_{dp},\left|{\dot{\tau }}_{d}^{q}\right|\leq k_{dq},\left|{\dot{\tau }}_{d}^{r}\right|\leq k_{dr}\forall t\geq 0$$

Since the first-order and second-order time derivatives of the desired attitude trajectory are bounded, Equation 13 is established.

(13) $$\left|{\dot{d}}_{11}\right|\leq k_{\phi },\left|{\dot{d}}_{12}\right|\leq k_{\theta },\left|{\dot{d}}_{13}\right|\leq k_{\varphi }$$

where $k_{\phi }>0,k_{\theta }>0,k_{\varphi }>0$.

Based on the mathematical formulation of $D_{1}$and in conjunction with Assumption 1, Equation 14 is derived.

(14) $$\left|{\dot{d}}_{21}\right|\leq k_{dp},\left|{\dot{d}}_{21}\right|\leq k_{dq},\left|{\dot{d}}_{21}\right|\leq k_{dr}$$

Let ${\hat{X}}_{1}$and ${\hat{D}}_{1}$ be the estimates of $X_{1}$and $D_{1}$ respectively. By defining ${\tilde{X}}_{1}=X_{1}-{\hat{X}}_{1}$, the following expression is derived

(15) $$\{\begin{array}{l} {\dot{\hat{X}}}_{1}=WX_{2}+{\hat{D}}_{1}\\ {\hat{D}}_{1}=\beta_{1}K_{1}{\tilde{X}}_{1}+\beta_{1}K_{2}\int_{0}^{t}{\tilde{X}}_{1}dt \end{array}$$

where


$$\beta_{1}=g_{1}+l_{1}\varphi (0,T_{1}),K_{1}=\left[\begin{array}{ccc} k_{11} & 0 & 0\\ 0 & k_{12} & 0\\ 0 & 0 & k_{13} \end{array}\right],K_{2}=\left[\begin{array}{ccc} k_{21} & 0 & 0\\ 0 & k_{22} & 0\\ 0 & 0 & k_{23} \end{array}\right]$$

Then Equation 16 holds.

(16) $${\dot{\tilde{X}}}_{1}={\dot{X}}_{1}-{\dot{\hat{X}}}_{1}=D_{1}-{\hat{D}}_{1}=D_{1}-\beta_{1}K_{1}{\tilde{X}}_{1}-\beta_{1}K_{2}\int_{0}^{t}{\tilde{X}}_{1}dt$$

Define $R_{1}=D_{1}-\beta_{1}K_{2}\int_{0}^{t}{\tilde{X}}_{1}dt$, Equation 17 can be obtained.

(17) $$\{\begin{array}{l} {\dot{\tilde{X}}}_{1}=-\beta_{1}K_{1}{\tilde{X}}_{1}+R_{1}\\ {\dot{R}}_{1}={\dot{D}}_{1}-\beta_{1}K_{2}{\tilde{X}}_{1} \end{array}$$

Define $e_{1}={\left[\begin{array}{cc} {\tilde{X}}_{1} & R_{1} \end{array}\right]}^{T}$ as the extended state vector of the system. The resulting dynamics are governed by the following equation

(18) $${\dot{e}}_{1}=\left[\begin{array}{c} -\beta_{1}K_{1}{\tilde{X}}_{1}+R_{1}\\ -\beta_{1}K_{2}{\tilde{X}}_{1}+{\dot{D}}_{1} \end{array}\right]=-\beta_{1}\left[\begin{array}{cc} K_{1} & B_{1}\\ K_{2} & 0 \end{array}\right]e_{1}+\left[\begin{array}{c} 0\\ {\dot{D}}_{1} \end{array}\right]$$

where


$$B_{1}=\left[\begin{array}{ccc} \frac{1}{-\beta_{1}} & 0 & 0\\ 0 & \frac{1}{-\beta_{1}} & 0\\ 0 & 0 & \frac{1}{-\beta_{1}} \end{array}\right]$$

By defining ${\mathbb{C}}_{1}=\left[\begin{array}{cc} K_{1} & B_{1}\\ K_{2} & 0 \end{array}\right],{\text{Δ}}_{1}=\left[\begin{array}{c} 0\\ {\dot{D}}_{1} \end{array}\right]$, the dynamic system described by Equation 18 reduces to the following compact form.

(19) $${\dot{e}}_{1}=-\beta_{1}{\mathbb{C}}_{1}e_{1}+{\text{Δ}}_{1}$$

Defining the Lyapunov function as Equation 20.

(20) $$V_{1}=\frac{1}{2}e_{1}^{T}e_{1}$$

Differentiating $V_{1}$ with respect to time and substituting the system dynamics from Equation 19 then Equation 21 holds.

(21) $$\begin{array}{c} {\dot{V}}_{1}=\frac{1}{2}{\dot{e}}_{1}^{T}e_{1}+\frac{1}{2}e_{1}^{T}{\dot{e}}_{1}\\ ={\dot{e}}_{1}^{T}e_{1}\\ ={\left(-\beta_{1}{\mathbb{C}}_{1}e_{1}+{\text{Δ}}_{1}\right)}^{T}e_{1}\\ =-\beta_{1}e_{1}^{T}{\mathbb{C}}_{1}^{T}e_{1}+{\text{Δ}}_{1}^{T}e_{1} \end{array}$$

Let $\lambda_{1}$ be the minimum eigenvalue of matrix ${\mathbb{C}}_{1}$, Equation 22 is established.

(22) $$\begin{array}{c} {\dot{V}}_{1}\leq -\beta_{1}\lambda_{1}{\left\|e_{1}\right\|}^{2}+\Delta_{1}^{T}e_{1}\\ \;\leq -\beta_{1}\lambda_{1}{\left\|e_{1}\right\|}^{2}+\left\|{\dot{D}}_{1}\right\|\left\|e_{1}\right\|\\ \;\leq -\beta_{1}{\left\|e_{1}\right\|}^{2}\left(\lambda_{1}-\frac{\left\|{\dot{D}}_{1}\right\|}{\beta_{1}\left\|e_{1}\right\|}\right) \end{array}$$

Defining the variable as in Equation 23.

(23) $$\lambda_{1}-\frac{\left\|{\dot{D}}_{1}\right\|}{\beta_{1}\left\|e_{1}\right\|}={\bar{h}}_{1}$$

Then Equation 24 holds.

(24) $$\begin{array}{c} {\dot{V}}_{1}\leq -\beta_{1}{\left\|e_{1}\right\|}^{2}{\bar{h}}_{1}\\ \;=-2{\bar{h}}_{1}\beta_{1}V_{1}\\ \;=-2{\bar{h}}_{1}\left(g_{1}+l_{1}\varphi (0,T_{1})\right)V_{1}\\ \;=-2{\bar{h}}_{1}V_{1}-2h_{1}l_{1}\varphi (0,T_{1})V_{1} \end{array}$$

Thus, through the proper selection of the design parameters $K_{1},K_{2},\beta_{1}$​, it can be ensured that ${\bar{h}}_{1}$ remains a positive real value. At this point, based on Lemma 1, the tracking error $e_{1}$ is guaranteed to converge to zero within the prescribed time $T_{1}$. As a result, the estimators ${\hat{X}}_{1}$ and ${\hat{D}}_{1}$ achieve an accurate estimation of the true states $X_{1}$​ and disturbances $D_{1}$​ within prescribed time.

Similarly, let ${\hat{X}}_{2}$and ${\hat{D}}_{2}$ be the estimates of $X_{2}$and $D_{2}$ respectively, By defining ${\tilde{X}}_{2}=X_{2}-{\hat{X}}_{2}$, the following expression is derived

(25) $$\{\begin{array}{l} {\dot{\hat{X}}}_{2}=F+J^{-1}\tau +{\hat{D}}_{2}\\ {\hat{D}}_{2}=\beta_{2}K_{3}{\tilde{X}}_{2}+\beta_{2}K_{4}\int_{0}^{t}{\tilde{X}}_{2}dt \end{array}$$

where


$$\beta_{2}=g_{2}+l_{2}\varphi (0,T_{2}),K_{3}=\left[\begin{array}{ccc} k_{31} & 0 & 0\\ 0 & k_{32} & 0\\ 0 & 0 & k_{33} \end{array}\right],K_{4}=\left[\begin{array}{ccc} k_{41} & 0 & 0\\ 0 & k_{42} & 0\\ 0 & 0 & k_{43} \end{array}\right]$$

Thus, through proper selection of the design parameters $K_{3},K_{4},\beta_{2}$, the estimators ${\hat{X}}_{2}$ and ${\hat{D}}_{2}$ achieve accurate estimation of the true states $X_{2}$​ and disturbances $D_{2}$​ within prescribed time. The stability proof which is similar to the previous analysis is omitted here to avoid redundancy.

### 3.2 Prescribed time backstepping sliding mode controller

In order to achieve prescribed time convergence of the attitude error dynamics, the controller must ensure prescribed time stability for both the sliding variables and the system states constrained to the sliding surface. The controller is systematically designed through the following steps.

Step 1: The first dynamic error surface is defined as

(26) $$S_{1}=X_{1}+\int_{0}^{t}\left(h_{11}X_{1}+h_{12}\varphi (T_{11},T_{12})X_{1}\right)dt$$

where $T_{11}$and $T_{12}$ denote the prescribed convergence time for the sliding surface and the system state variables, respectively. Defining a variable coefficient expon $T_{12}$ntial approach law as follows

(27) $${\dot{S}}_{1}=-\left(\alpha_{11}+\alpha_{12}\varphi (T_{21},T_{11})\right)S_{1}-\eta_{1}sign(S_{1})$$

By taking the time derivative of Equation 26 and incorporating the dynamics from Equations 6, 27, the virtual control input is derived as follows

(28) $$X_{2}^{c}=-W^{-1}\left(\left(\alpha_{11}+\alpha_{12}\varphi (T_{21},T_{11})\right)S_{1}+\eta_{1}sign(S_{1})+\left(h_{11}X_{1}+h_{12}\varphi (T_{11},T_{12})X_{1}\right)+{\hat{D}}_{1}\right)$$

Step 2: The second dynamic error surface is defined as

(29) $$S_{2}=X_{2}-X_{2}^{c}$$

Defining a variable coefficient exponential approach law as Equation 30.

(30) $${\dot{S}}_{2}=-\left(\alpha_{21}+\alpha_{22}\varphi (0,T_{21})\right)S_{2}-\eta_{2}sign(S_{2})$$

Then, the following controller can be obtained

(31) $$\tau =-J\left(\left(\alpha_{21}+\alpha_{22}\varphi (0,T_{21})\right)S_{2}+\eta_{2}sign(S_{2})+{\hat{D}}_{2}+F-{\dot{X}}_{2}^{c}\right)$$

Theorem 1. The attitude system of plant-protection UAVs is regarded as a nonlinear system, and the attitude tracking error dynamics is described by Equation 6, where the exogenous disturbances satisfy the conditions specified in Assumption 1. When the extended state observers formulated in Equations 15, 25 are utilized to estimate the system errors $D_{1}$ and $D_{2}$, and the prescribed time backstepping sliding mode control in Equation 32 is applied, then the closed-loop system guarantees that all attitude angle tracking errors converge to zero within a prescribed time $T_{12}$.

(32) $$\{\begin{array}{l} S_{1}=X_{1}+\int_{0}^{t}\left(h_{11}X_{1}+h_{12}\varphi (T_{11},T_{12})X_{1}\right)dt\\ X_{2}^{c}=-W^{-1}\left(\left(\alpha_{11}+\alpha_{12}\varphi (0,T_{11})\right)S_{1}+\eta_{1}sign(S_{1})+\left(h_{11}X_{1}+h_{12}\varphi (T_{11},T_{12})X_{1}\right)+{\hat{D}}_{1}\right)\\ S_{2}=X_{2}-X_{2}^{c}\\ \tau =-J\left(\left(\alpha_{21}+\alpha_{22}\varphi (0,T_{21})\right)S_{2}+\eta_{2}sign(S_{2})+{\hat{D}}_{2}+F-{\dot{X}}_{2}^{c}\right) \end{array}$$

### 3.3 Stability analysis

The stability analysis for the proposed control algorithm proceeds through the following systematic procedure.

Step 1: Demonstrate that under the control law specified in Equation 31, the system state $X_{2}$ achieves exact tracking of the virtual control $X_{2}^{c}$ input within prescribed time, which implies the prescribed time convergence of the sliding mode variable.

Defining the Lyapunov function as Equation 33.

(33) $$V_{21}=\frac{1}{2}S_{2}^{T}S_{2}$$

Differentiating $V_{21}$ with respect to time and substituting the derivative of Equation 29, along with Equations 31, 6, yield Equation 34.

(34) $$\begin{array}{c} {\dot{V}}_{21}=\frac{1}{2}{\dot{S}}_{2}^{T}S_{2}+\frac{1}{2}S_{2}^{T}{\dot{S}}_{2}\\ ={\dot{S}}_{2}^{T}S_{2}\\ ={\left(F+J^{-1}\tau +D_{2}-{\dot{X}}_{2}^{c}\right)}^{T}S_{2}\\ ={\left(-\left(\alpha_{21}+\alpha_{22}\varphi (0,T_{21})\right)S_{2}-\eta_{2}sign(S_{2})+D_{2}-{\hat{D}}_{2}\right)}^{T}S_{2} \end{array}$$

From the previous derivation, the variable ${\hat{D}}_{2}$ achieves accurate estimation of $D_{2}$ within prescribed time. Defining $e_{d2}$ as the supremum of the minimal upper bounds on the estimation errors of the external disturbance torques along the three body-fixed axes and by choosing a suitable sign function gain $\eta_{2}$ to satisfy condition $\eta_{2}\geq \left|e_{d2}\right|$, Equation 35 is established

(35) $$\begin{array}{c} {\dot{V}}_{21}\leq -\left(\alpha_{21}+\alpha_{22}\varphi (0,T_{21})\right)S_{2}^{T}S_{2}-(\eta_{2}-e_{d2})||S_{2}||\\ \leq -\alpha_{21}V_{21}-\alpha_{22}\varphi (0,T_{21})V_{21} \end{array}$$

Based on Lemma 1, the sliding mode variable $S_{2}$ converges within prescribed time, which implies the system state $X_{2}$ achieves exact tracking of the virtual control $X_{2}^{c}$ input within prescribed time.

Step 2: Demonstrate that under the virtual control law specified in Equation 28, the sliding variable $S_{1}$ achieves prescribed-time convergence.

Defining the Lyapunov function as Equation 36.

(36) $$V_{11}=\frac{1}{2}S_{1}^{T}S_{1}$$

Differentiating $V_{11}$ with respect to time and substituting the derivative of Equation 26, along with Equations 28, 6, yields Equation 37.

(37) $$\begin{array}{c} {\dot{V}}_{11}=\frac{1}{2}{\dot{S}}_{1}^{T}S_{1}+\frac{1}{2}S_{1}^{T}{\dot{S}}_{1}\\ ={\dot{S}}_{1}^{T}S_{1}\\ ={\left({\dot{X}}_{1}+\left(h_{11}X_{1}+h_{12}\varphi (T_{11},T_{12})X_{1}\right)\right)}^{T}S_{1}\\ ={\left(WX_{2}^{c}+D_{1}+\left(h_{11}X_{1}+h_{12}\varphi (T_{11},T_{12})X_{1}\right)\right)}^{T}S_{1}\\ ={\left(-\left(\alpha_{11}+\alpha_{12}\varphi (T_{21},T_{11})\right)S_{1}-\eta_{1}sign(S_{1})-{\hat{D}}_{1}+D_{1}\right)}^{T}S_{1} \end{array}$$

From the previous derivation, the variable ${\hat{D}}_{1}$ achieves accurate estimation of $D_{1}$ within prescribed time. Defining $e_{d1}$ as the supremum of the minimal upper bounds on the ${\hat{D}}_{1}$ estimation error vector for the first-order time derivatives of the three attitude angles and by choosing a suitable sign function gain $\eta_{1}$ to satisfy condition $\eta_{2}\geq \left|e_{d2}\right|$, Equation 38 is established

(38) $$\begin{array}{c} {\dot{V}}_{11}\leq -\left(\alpha_{11}+\alpha_{12}\varphi (T_{21},T_{11})\right)S_{1}^{T}S_{1}-(\eta_{1}-e_{d1})\left||S_{1}|\right|\\ \leq -\alpha_{11}V_{11}-\alpha_{12}\varphi (T_{21},T_{11})V_{11} \end{array}$$

Based on Lemma 1, the state variables $X_{1}$ reach the sliding surface $S_{1}$ within prescribed time $T_{11}$, which ensures prescribed-time stability of the sliding variables.

Step 3: Demonstrate the convergence of system states $X_{1}$ within prescribed time.

After the sliding mode variable $S_{1}$ converges within prescribed time, the state variables $X_{1}$ will remain on the sliding mold surface, i.e., $S_{1}={\dot{S}}_{1}=0$, and the time of this process satisfies $t\geq T_{11}$. Defining the Lyapunov function as follows

(39) $$V_{12}=\frac{1}{2}X_{1}^{T}X_{1}$$

From Equation 26, the following relation is derived

(40) $$\begin{array}{c} {\dot{V}}_{12}=\frac{1}{2}{\dot{X}}_{1}^{T}X_{1}+\frac{1}{2}X_{1}^{T}{\dot{X}}_{1}\\ ={\dot{X}}_{1}^{T}X_{1}\\ =-{\left(h_{11}X_{1}+h_{12}\varphi (T_{11},T_{12})X_{1}\right)}^{T}X_{1}\\ =-h_{11}X_{1}^{T}X_{1}-h_{12}\varphi (T_{11},T_{12})X_{1}^{T}X_{1}\\ =-h_{11}V_{12}-h_{12}\varphi (T_{11},T_{12})V_{12} \end{array}$$

Taking the time derivative of Equation 39 and incorporating the derivative relation from Equation 40, we obtain Equation 41.

(41) $$\begin{array}{c} {\dot{V}}_{12}=\frac{1}{2}{\dot{X}}_{1}^{T}X_{1}+\frac{1}{2}X_{1}^{T}{\dot{X}}_{1}\\ ={\dot{X}}_{1}^{T}X_{1}\\ =-{\left(h_{11}X_{1}+h_{12}\varphi (T_{11},T_{12})X_{1}\right)}^{T}X_{1}\\ =-h_{11}X_{1}^{T}X_{1}-h_{12}\varphi (T_{11},T_{12})X_{1}^{T}X_{1}\\ =-h_{11}V_{12}-h_{12}\varphi (T_{11},T_{12})V_{12} \end{array}$$

Based on Lemma 1, the state vector $X_{1}$ achieves convergence within prescribed time $T_{12}$. The stability analysis of the proposed algorithm is now formally established.

## 4 Numerical simulations

In order to validate the feasibility and efficacy of the proposed algorithm, this section takes the attitude system model of a quadrotor sprayer UAV as an example. First, the prescribed time convergence performance of the proposed control method under different initial conditions is validated. Then, comparative experiments are conducted to demonstrate the effectiveness of the proposed algorithm. The specific parameters of the UAV are as follows: $J_{X}=J_{Y}=1.089\times 10^{-2}kg\cdot m^{2},J_{Z}=2.178\times 10^{-1}kg\cdot m^{2},m=12kg,l=0.825m,b=2\times 10^{-6},K_{L}=8.42\times 10^{-5}$. The attitude angle tracking command is set to the following time-varying form: $\phi^{d}=-10^{\circ }+20^{\circ }\cos(\pi t/2),\theta^{d}=-15^{\circ }\cos(\pi t/2),\varphi^{d}=0$. The exogenous disturbances acting on the system are specified as follows: $\tau_{d}^{p}=-4-1.2\sin(\pi t/8),\tau_{d}^{q}=3+1.2\sin(\pi t/8)$, $\tau_{d}^{r}=-3-1.2\sin(\pi t/8)$. The controller parameters are selected as follows: $p=5,g_{1}=g_{2}=2,l_{1}=l_{2}=1,T_{1}=0.5s,T_{2}=0.3s,\eta_{1}=\eta_{2}=0.1$, $k_{11}=k_{12}=k_{13}=k_{31}=k_{32}=k_{33}=2,k_{21}=k_{22}=k_{23}=k_{41}=k_{42}=k_{43}=0.1,h_{11}=0.6,h_{12}=2\alpha_{11}=0.25,\alpha_{12}=0.4$, $\alpha_{21}=2,\alpha_{22}=3.2,T_{11}=1s,T_{12}=2s,T_{21}=0.4s$.

**Figure 2:**
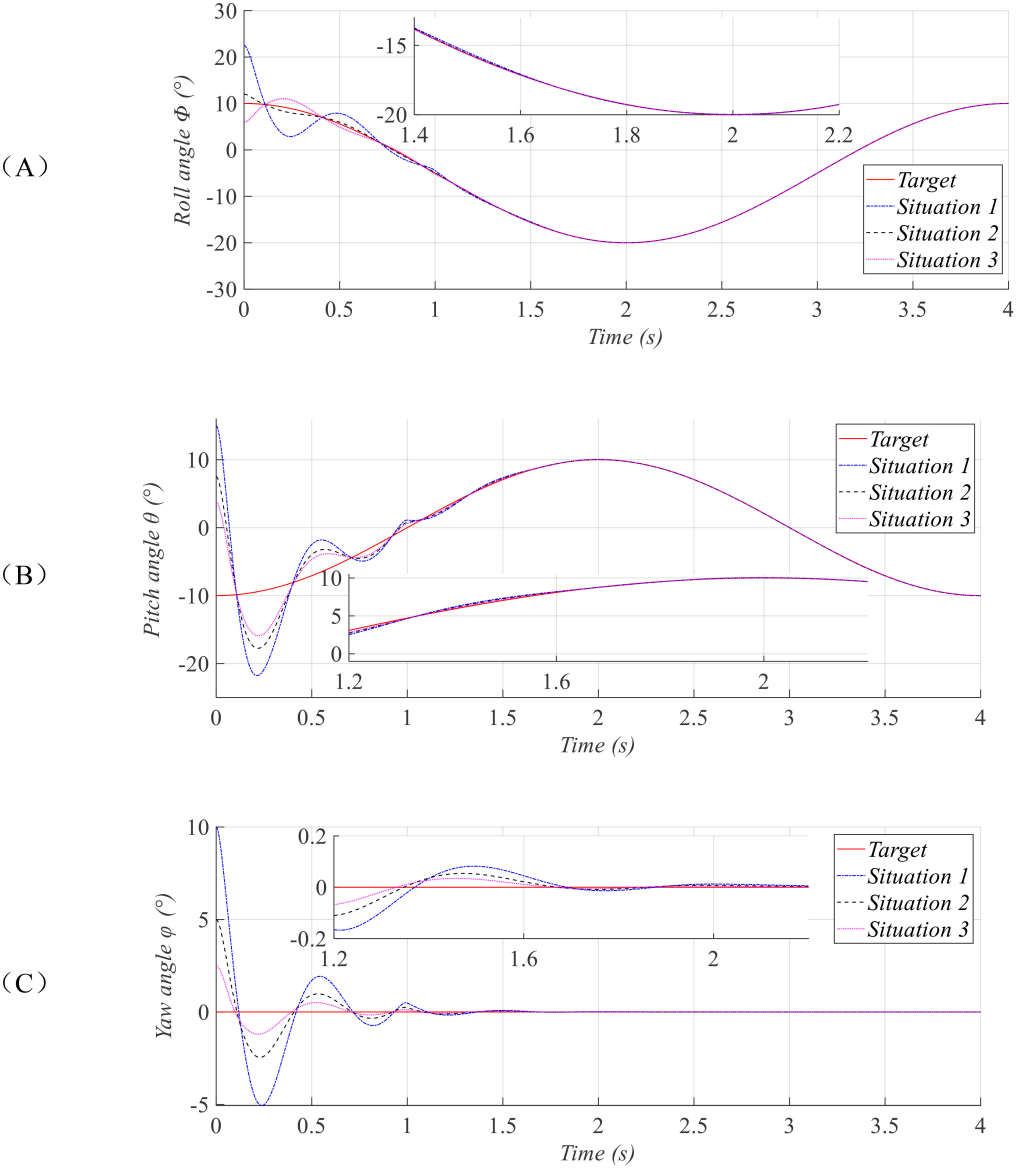
Tracking curves of the attitude angles: roll **(A)**, pitch **(B)**, and yaw **(C)**.

**Figure 3:**
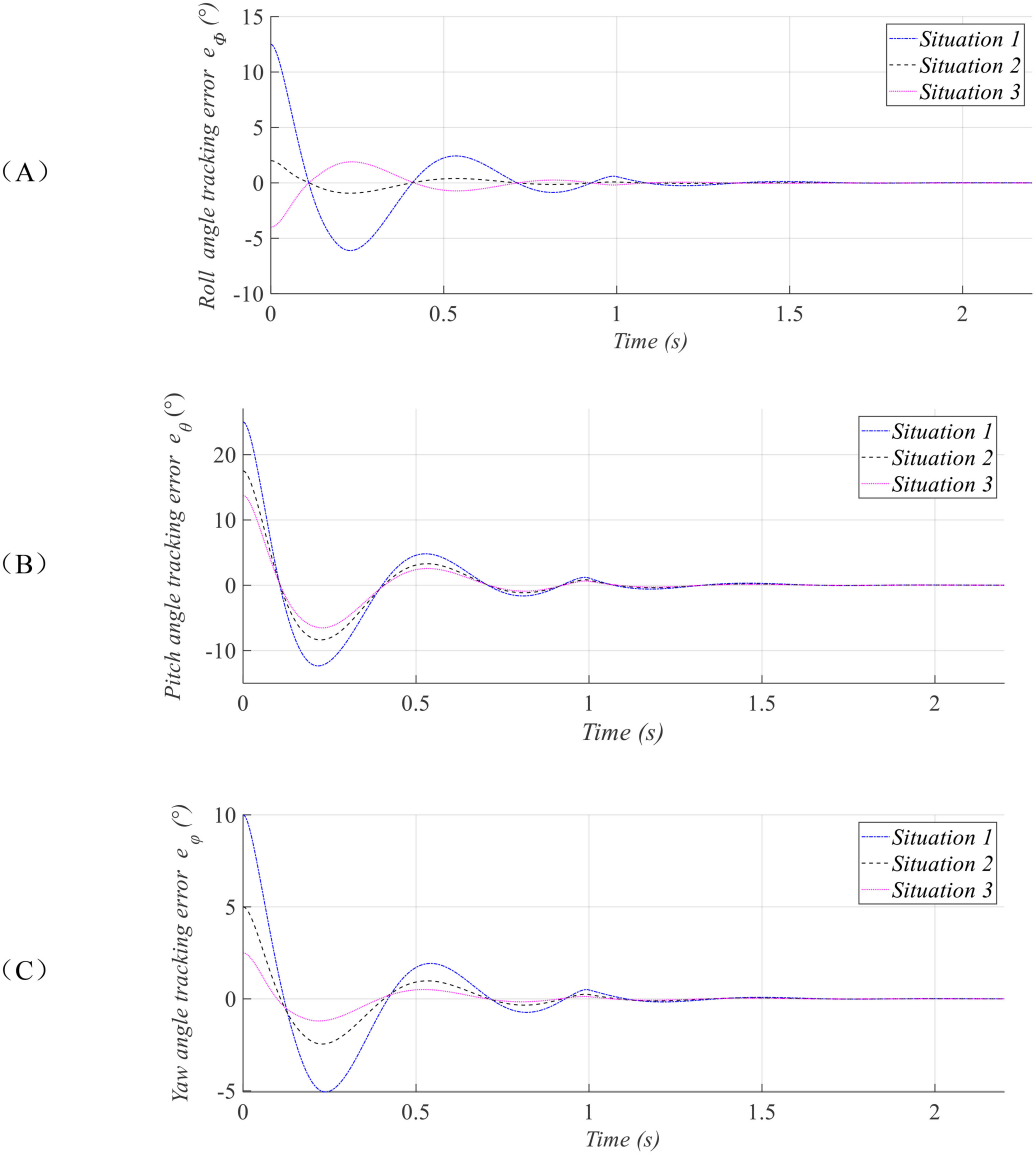
Tracking error curves of the attitude angles: roll **(A)**, pitch **(B)**, and yaw **(C)**.

**Figure 4:**
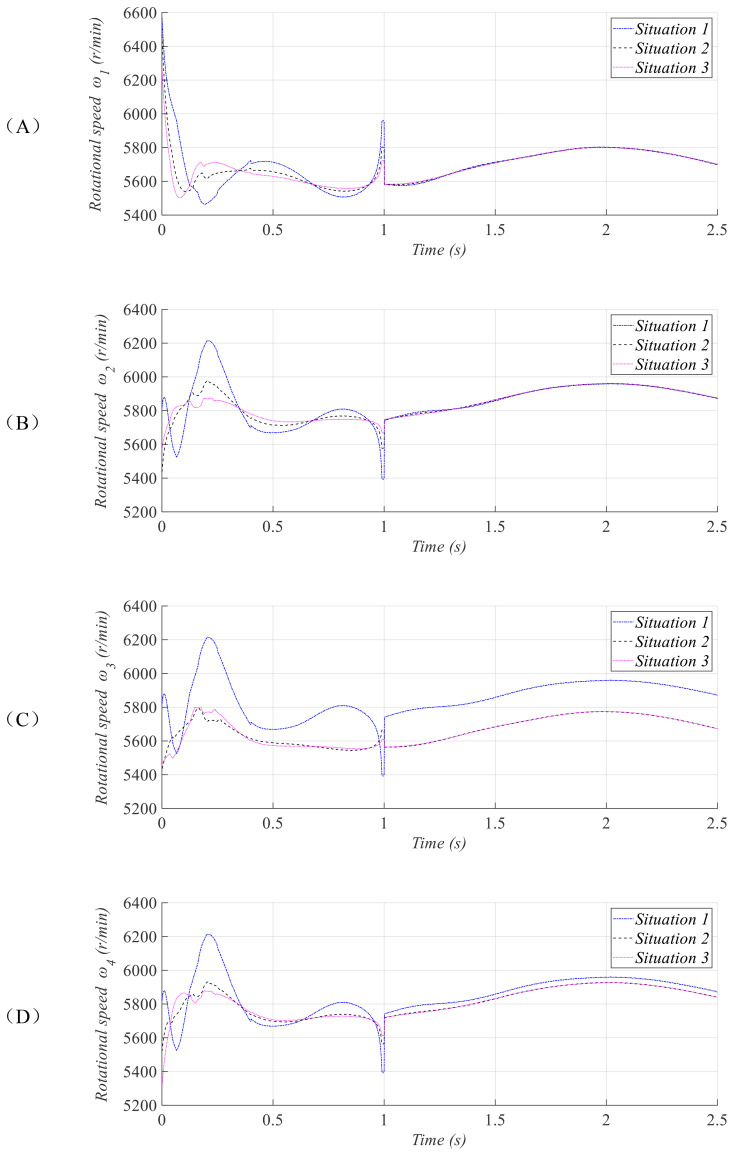
Rotational speed curves of rotors 1 **(A)**, 2 **(B)**, 3 **(C)**, and 4 **(D)**.

Three different sets of initial attitude conditions are selected for simulation studies. The first set is specified as ϕ=22.5π/180,θ=π/12,φ=π/18, the second set is ϕ=12π/180,θ=π/24,φ=π/36, th ϕ=12π/180,θ=π/24,φ=π/36 third set is ϕ=π/30,θ=π/48,φ=π/72. The simulation results are presented in **Figures 2**–**4**.

**Figures 2**, **3** depict the attitude tracking performance and corresponding tracking error responses of the agricultural UAV under three distinct initial conditions. The results demonstrate that although the initial attitude angle of the of plant-protection UAV varies greatly in the three situations, they can all converge within the prescribed time $T_{12}$, which is not affected by the initial state. This ensures that when the UAV is disturbed and deviates from its predetermined attitude, it can return to the stable state within the same time. The UAV can adjust the spraying speed according to this preset time, achieving uniform spraying.

**Figure 4** presents the rotational speed curves of the quadrotor for the UAV under three initial conditions. It can be observed that different initial conditions only result in rotational speed differences during the initial phase, and all cases can maintain stable and consistent rotational speeds within the time $T_{12}$. As can be observed, the rotational speeds of the four rotors remain at a similar level, with their energy requirements being largely consistent. This contributes to the stabilization of the drone’s attitude.

In order to demonstrate the efficacy of the proposed control strategy, comparative experiments are performed against the fast sliding mode control (FSMC) which uses the fast power-reaching law proposed by Han et al. (2025). The sliding mode surface for the benchmark controller is designed as Equation 42.

(42) $$S=\eta X_{2}+X_{1}$$

The control law is formulated as Equation 43

(43) $$\tau =\frac{1}{\eta }(-k_{1}S-K_{2}sign^{\rho }(S)-X_{2})-\hat{D}-F$$

where $k_{1}=3,k_{2}=20,\rho =0.6,\eta =0.2$.

**Figure 5:**
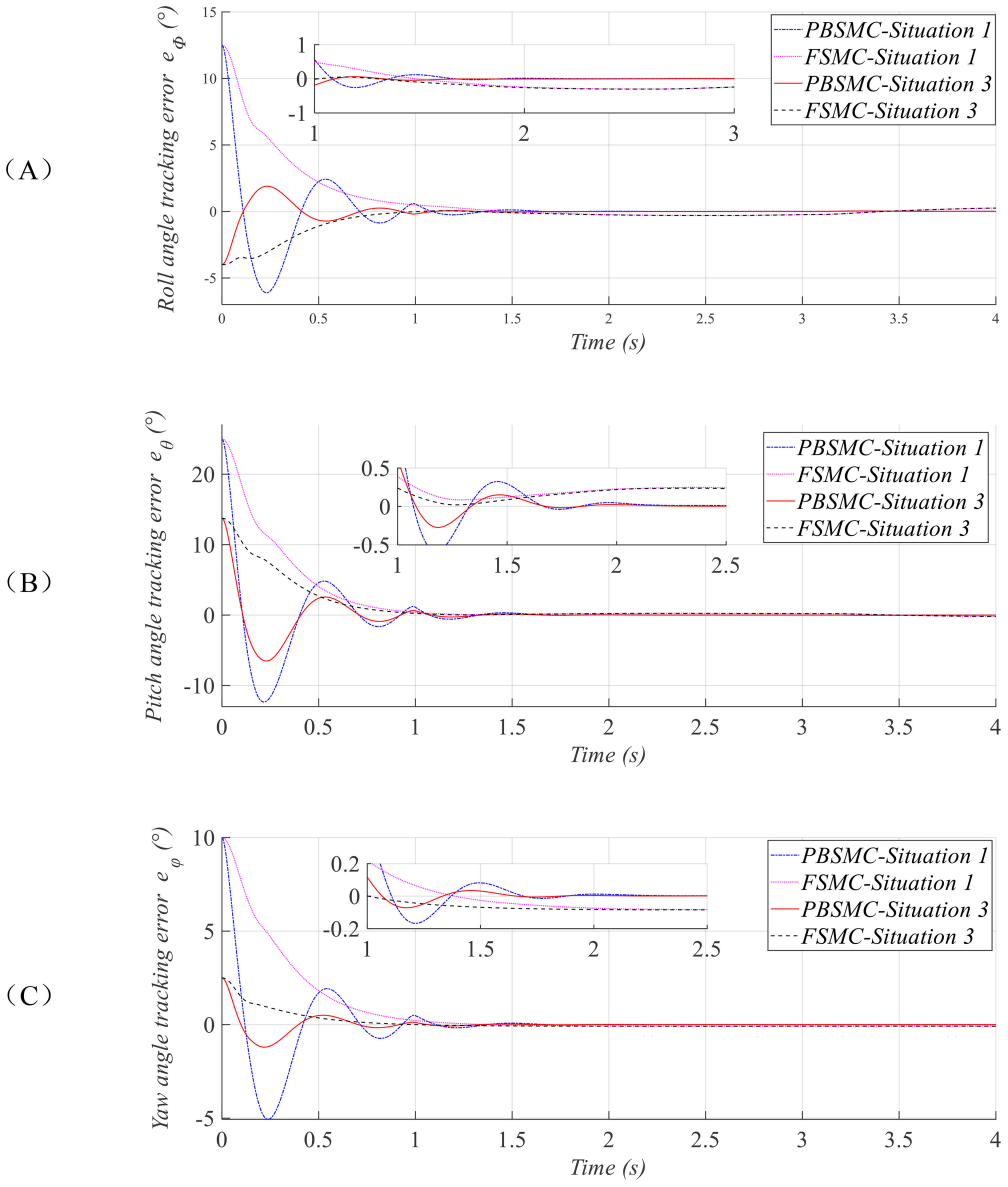
Comparison curves of attitude angle tracking errors in the pitch **(A)**, yaw **(B)**, and roll **(C)** channels.

A comparative analysis is conducted using the first and third sets of initial conditions, and the corresponding simulation results are presented in **Figure 5**.

**Figure 5** illustrates the attitude tracking error responses under the first and third initial condition sets. As observed in the partially enlarged detail, under both test conditions, the proposed PBSMC achieves convergence of attitude errors to a minimal range within the specified time frame. In contrast, the FSMC used for comparison demonstrates oscillatory attitude angle errors within a substantially larger margin.

## 5 Conclusion

This paper addresses the attitude control system of plant-protection UAVs during spraying operations, investigating the attitude command tracking control problem under strong wind gusts and motor dynamic disturbances. A prescribed time backstepping sliding mode control method is proposed. Numerical simulation results demonstrate that the proposed method can achieve attitude stability in preset time, which prevents the attitude stabilization from being affected by the system’s initial conditions and provides a guarantee for the uniform spraying of the plant-protection UAVs.

However, the verification in this paper is limited to numerical simulations and does not incorporate many real-world factors of spray drones in field operations. Thus, in future research, the authors will consider the impact of payload variations and liquid sloshing on sprayer UAVs and further develop robust attitude control methodologies for agricultural spraying applications. In addition, the authors will also conduct hardware-in-the-loop tests and experimental UAV flight trials to enhance the credibility of the theory and promote the transformation from theory to practice.

## Data Availability

The original contributions presented in the study are included in the article/supplementary material. Further inquiries can be directed to the corresponding author.
